# Identification of Omicron-Delta Coinfections Using PCR-Based Genotyping

**DOI:** 10.1128/spectrum.00605-22

**Published:** 2022-05-03

**Authors:** Pavitra Roychoudhury, Shishi Luo, Kathleen Hayashibara, Pooneh Hajian, Margaret G. Mills, Jean Lozach, Tyler Cassens, Seffir T. Wendm, Isabel Arnould, David Becker, Tim Wesselman, Jeremy Davis-Turak, Richard Creager, Eric Lai, Keith R. Jerome, Tracy Basler, Andrew Dei Rossi, William Lee, Alexander L. Greninger

**Affiliations:** a Department of Laboratory Medicine and Pathology, University of Washington, Seattle, Washington, USA; b Vaccine and Infectious Disease Division, Fred Hutchinson Cancer Research Center, Seattle, Washington, USA; c Helix, San Mateo, California, USA; d Thermo Fisher Scientific, South San Francisco, California, USA; e Rosalind Bio, San Diego, California, USA; f NaviDx, LLC, Newport Beach, California, USA; g Personalized Science, LLC, South Burlington, Vermont, USA; Barnard College, Columbia University

**Keywords:** Delta, Omicron, PCR genotyping, SARS-CoV-2, WGS, coinfection, ddPCR, genomics, variants

## LETTER

The Omicron variant of SARS-CoV-2 has driven an explosion of cases in many parts of the world due to its high transmissibility and ability to evade preexisting immunity (1). Prior to the arrival of the Omicron variant in late November 2021, the Delta variant constituted >99% of all positive samples sequenced in the United States, where about 100,000 cases were being reported daily. Here we report the first cases of Omicron-Delta mixed infection identified in four samples using a PCR-based genotyping panel. For two of these samples, the mixed infection was confirmed with RT-droplet digital PCR (RT-ddPCR) and two separate amplicon-based sequencing approaches. For the other two samples, which were identified independently at a commercial lab, the mixed infection was confirmed via two separate rounds of hybrid-capture sequencing.

**FIG 1:**
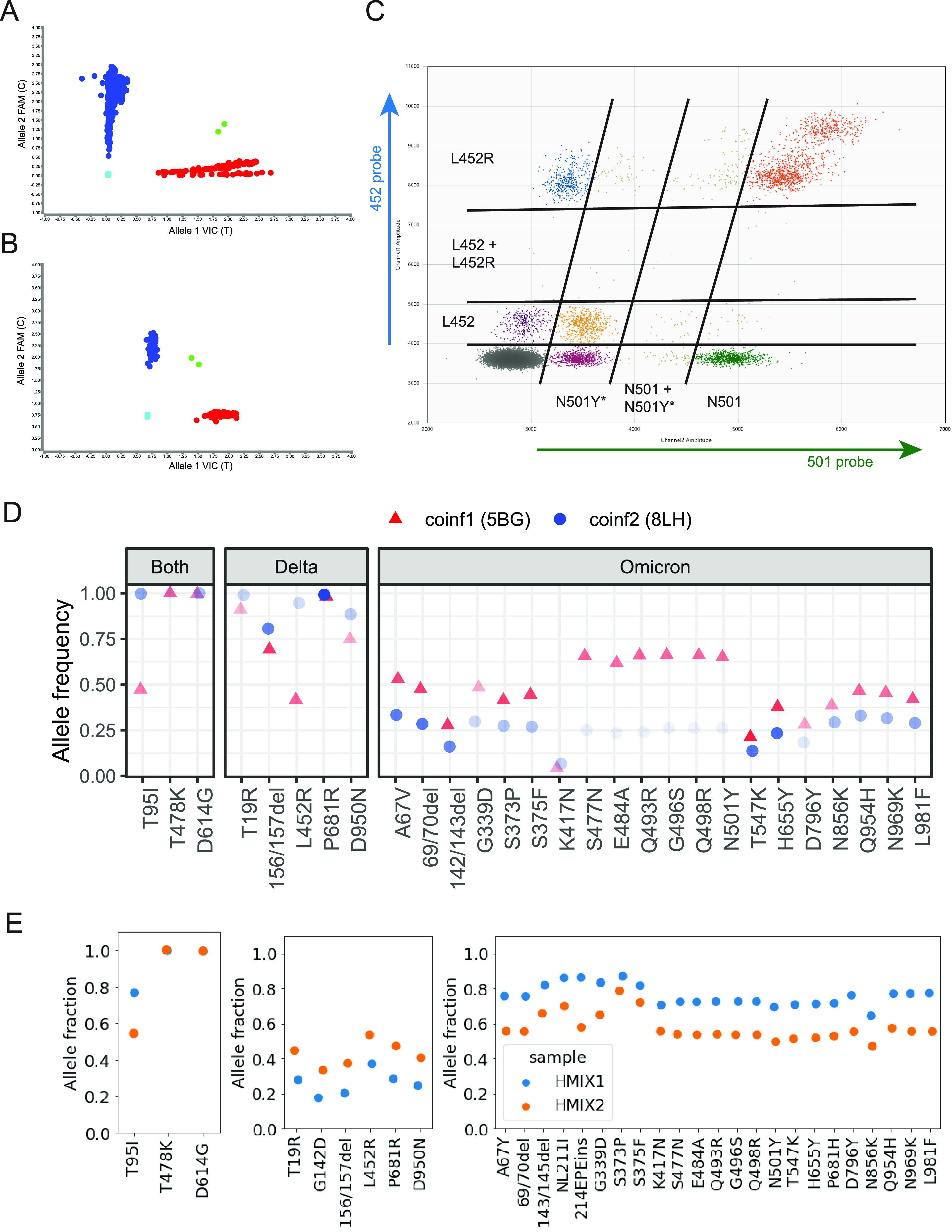
Identification of Delta-Omicron coinfection. Allele-specific PCR targeting SARS-CoV-2 allele T13195C of plates containing omicron (T13195C, blue) and delta (T13195, red) infections as well as two co-infection specimens from UW (A) and Helix (B). Mixed samples are clustered in green in the middle of the plot. NTCs are clustered in light blue and are oriented to the origin. (C) RT-ddPCR for coinfection sample 5BG showing amplification of both 452 alleles (L = Omicron; R = Delta) and both 501 alleles (Y* = Omicron, N = Delta) from UW. (D) Allele frequency of signature spike gene mutations commonly of Omicron or Delta or both from deep sequencing of two coinfection samples from UW shows presence of minor alleles corresponding to Omicron and (E) similarly from Helix. Transparency of plotting characters is inversely proportional to depth of coverage at the site in Fig. 1D.

RNA was extracted from randomly selected samples positive for SARS-CoV-2 by RT-qPCR with a cycle threshold (Ct) value <= 33. Allele-specific PCR was performed to detect 4 targets designed to distinguish between Omicron and Delta infection (G8393A (ORF1ab:A2710T), T13195C, C21618G (S:T19R), C23202A (S:T547K)). In four samples (5BG, 8LH, HMIX1, and HMIX2; Table S1) out of more than 10,000 positives screened (2), intermediate levels of amplification were detected for both alleles on all 4 targets suggesting mixed infection (Fig. 1A). Individual 5BG was vaccinated with a booster and reported 3 days of unspecified symptoms after contact with a known positive and 8LH reported ageusia and no other information. No clinical information was available for HMIX1 and HMIX2. Samples 5BG and 8LH were also tested by a separate PCR assay to detect S-gene target failure (ThermoFisher TaqPath assay), and a droplet digital (RT-dd)PCR assay targeting four different loci in the spike gene (417K, 452L, 484E, 501N) (3, 4) (Table S1, Fig. 1B). Viral whole genome sequencing was performed on both coinfection samples using the Swift SNAP amplicon panel (IDT) (5, 6). Although the resulting consensus genomes classified as Delta lineages (AY.25 and AY.4) (7), and SGTF showed no S-dropout, the presence of minor alleles corresponding to signature Omicron mutations at frequencies between 5 and 40% was consistent with mixed infection (Fig. 1C). In addition, RT-ddPCR assay results showed droplets corresponding to both alleles at each of the four target loci at frequencies between 18 and 34% (Fig. 1B, Fig. S2 in the supplemental material). All results replicated on repeat extraction and testing of the specimens. Samples HMIX1 and HMIX2 were identified in a commercial laboratory running the same allele-specific PCR. These two samples were sequenced and re-sequenced via a hybrid-capture assay to confirm allele fractions strongly suggestive of a mixture of Delta and Omicron variants (Fig. 1D and Supplemental Methods).

Overall, our results confirm the presence of both Omicron and Delta variants in four samples taken from different individuals in two different laboratories, suggesting coinfection. We specifically targeted specimens with high minor allele frequencies (>10%) that could be readily discernible by sequencing. We ruled out contamination using repeat extractions from the original specimens, and resequencing for one sample using a different amplicon panel showed similar allele frequencies (Illumina COVIDseq, Fig. S1 in the supplemental material). A limitation of the approach is that we did not recollect the individuals and thus cannot entirely rule out pre-analytical contamination. Given high levels of community spread, mixed infections may be more prevalent, but cannot be easily identified from consensus genomes without additional analysis. Our results therefore highlight another use-case for qPCR genotyping of suspected coinfections for situations where a more rapid turnaround may be required for clinical decision-making (8) as well as potential concern for inter-variant SARS-CoV-2 recombination due to co-infection.
