# Supplementary material for: Identification of Omicron-Delta Coinfections Using PCR-Based Genotyping
Source: Microbiol Spectr. 2022 May 3;10(3):e00605-22. doi: 10.1128/spectrum.00605-22 (PMC9241779; doi:10.1128/spectrum.00605-22)
Supplement: SUPPLEMENTAL FILE 1 — Supplemental material. Download spectrum.00605-22-s001.pdf, PDF file, 0.1 MB [file spectrum.00605-22-s001.pdf]

Supplemental information

Sample selection and nucleic acid extraction

Clinical specimens used in this study included nasopharyngeal, oropharyngeal, or nasal swabs that were tested by PCR for SARS-CoV-2 at the University of Washington Virology Lab. Samples with a Ct value of 33 or lower were retained for PCR testing and sequencing. The use of residual clinical specimens for genotyping and sequencing was approved by the University of Washington Institutional Review Board with a waiver of informed consent. Total nucleic acids were extracted using either ThermoFisher KingFisher or Roche MagNA Pure 96 instruments according to manufacturer instructions. All extractions used 200µl of input volume and 50µl elution.

PCR-based genotyping for variant identification

PCR-based variant identification was performed using the TaqMan SARS-CoV-2 Mutation Panel (ThermoFisher) following manufacturer instructions. The reaction mix for each of 4 assays (G8393A_ANYM2N3_, T13195C_ANZTV9Z_, C23202A_ANFV3TP_S.T547K, C21618G_ANCFJNG_S.T19R) containing primers and probes to detect reference and mutant alleles was combined with TaqPath 1-Step RT-qPCR Master Mix CG (ThermoFisher). For each assay, 384-well plates were prepared with each well containing 2.5 µl of extracted RNA and 7.5 µl of reaction mix using the Microlab VANTAGE 2.0 liquid Handling System (Hamilton). Plates were sealed using MicroAmp Optical Adhesive Film (ThermoFisher), vortexed and centrifuged. Real-time RT-PCR was performed using QuantStudio7 instruments (ThermoFisher).

RT-ddPCR

RNA from suspected coinfection specimens was diluted to a target Ct value of ~28 to maximize the probability that droplets contain at most one copy of viral RNA. Reverse transcription-droplet digital (RT-dd)PCR was carried out as described previously [4], using three duplex reactions to identify mutations in codons for Spike amino acids 417, 452, 484, and 501. RNA from known Delta (417K, L452R, 484E or E484Q, 501N) and Omicron (K417N, 452L, E484A, G496S/Q498R/N501Y) specimens were included as positive controls in each RT-ddPCR run.

Spike gene target failure

Spike gene target failure was detected using the TaqPath PCR assay (ThermoFisher) performed according to manufacturer's instructions. Four 96-well plates were combined into a single 384-well plate using the Microlab VANTAGE 2.0 liquid Handling System (Hamilton), with each well containing 10 µl of extracted RNA and 10 µl of reaction mix (TaqPath RT-PCR COVID-19 Kit, ThermoFisher). Plates were sealed using MicroAmp Optical Adhesive Film (ThermoFisher), vortexed and centrifuged. Real-time RT-PCR was performed using QuantStudio7 instruments (ThermoFisher). Spike gene target failure was determined to have occurred if there was no detectable amplification of the S target, assessed using multicomponent plots.

Viral genome sequencing and analysis

Viral genome sequencing was performed as described previously [5]. Briefly, RNA was extracted from residual clinical specimens using either Roche MagNA Pure 96 or ThermoFisher KingFisher platforms following manufacturer instructions. Sequencing libraries were prepared using

multiplexed amplicon panels from Swift Biosciences (IDT) or Illumina COVIDSeq and sequenced on Illumina NextSeq 2000 or NovaSeq instruments using a 2x150 read format for Swift, and 1x100 format for COVIDSeq libraries. Raw reads were processed using a custom bioinformatics pipeline (https://github.com/greninger-lab/covid_swift_pipeline) that performs adapter and quality trimming and filtering, masking of primers, generation of consensus sequences, and variant annotation. Phylogenetic lineage assignment was done using Pangolin (https://pangolin.cog-uk.io/).

Hybrid capture based viral genome sequencing and analysis

Remnant specimens that tested positive for SARS-CoV-2 by qPCR diagnostic tests were subjected to RNA extraction, followed by RNA library preparation, and then SARS-CoV-2 genome capture using IDT xGen COVID-19 Capture Panel. Next-generation sequencing using the NovaSeq Sequencing system with 2-150bp reads was performed. After demultiplexing, reads were processed using the Helix fastagenerator pipeline (as described in https://papers.ssrn.com/sol3/papers.cfm?abstract_id=3952083) to generate a consensus sequence as well as variant call format (VCF) files.
